# Establishment and application of a reverse dot blot assay for 13 mutations of hearing-loss genes in primary hospitals in China

**DOI:** 10.2478/abm-2024-0003

**Published:** 2024-03-20

**Authors:** Qing-Qing Jiang, Juan-Juan Zhu, Shu-Ling Fan, Ya-Ping Hou, Xie-Ying Hu, Jie Shi, Lei Wu, Ying Luo

**Affiliations:** Department of Clinical Laboratory, Changning Maternity and Infant Health Hospital, East China Normal University, Shanghai 200050, China; Chaozhou Hybribio Limited Corporation, Guangdong, China; Shanghai Tenth People's Hospital, Tongji University School of Medicine, Shanghai 200072, China; Nanjing Red Cross Blood Center, Nanjing, Jiangsu 210037, China

**Keywords:** *GJB2*, *GJB3*, hearing-loss gene, *MT-RNR1*, reverse dot blot assay, *SLC26A4*

## Abstract

**Background:**

Hearing loss is a common sensorineural dysfunction with a high incidence in China. Although genetic factors are important causes of hearing loss, hearing-related gene detection has not been widely adopted in China.

**Objective:**

Establishing a rapid and efficient method to simultaneously detect hotspot hearing loss gene mutations.

**Methods:**

A reverse dot blot assay combined with a flow-through hybridization technique was developed for the simultaneous detection of 13 hotspot mutations of 4 hearing loss–related genes including *GJB2, GJB3, SLC26A4,* and the mitochondrial gene *MT-RNR1*. This method involved PCR amplification systems and a hybridization platform.

**Results:**

The technique can detect 13 hotspot mutations of 4 hearing loss–related genes. And a total of 213 blood samples were used to evaluate the availability of this method.

**Discussion:**

Our reverse dot blot assay was a simple, rapid, accurate, and cost-effective method to identify hotspot mutations of 4 hearing loss–related genes in a Chinese population.

Hearing loss is a highly prevalent congenital disease that severely affects the quality of patients' daily life. It is estimated that about 466 million people (5.5% of the population) have disabling hearing loss worldwide (https://www.who.int/health-topics/hearing-loss). In the Chinese population, there are about 0.8 million children under the age of 7 suffering from impairment of hearing, and the proportion shows an increase of 30,000 new cases per year [1, 2]. The etiology of hearing loss is heterogeneous. Approximately 60% of hearing loss cases are found to be attributed to genetic causes [3]. So far, 124 non-syndromic hearing loss (NSHL) genes (including 51 identified autosomal dominant genes, 77 autosomal recessive genes, and 5 X-linked genes) (https://hereditaryhearingloss.org) have been reported.

In the Chinese population, nearly 50% of cases of NSHL are associated with mutations in the following genes: *GJB2* (OMIM: 121011), *GJB3* (OMIM: 603324), *SLC26A4* (OMIM: 605646) and the mitochondrial gene *MT-RNR1* (OMIM: 561000) [4]. The mutation frequencies of *GJB2*, *GJB3*, *SLC26A4,* and *MT-RNR1* in Chinese NSHL patients are 27.13%, 2.01%, 20.85%, and 2.26%, respectively [5]. The five-most common mutations (c.35delG, c.155_158del4, c.176_191del16, c.235delC, and c.299_300delAT) of *GJB2* are associated with congenital deafness susceptibility in the Chinese population [6, 7, 8]. c.235delC, which is the most prevalently observed pathogenic mutation accounting for 88.0% of all mutant sites identified [9, 10]. Similarly, in the *SLC26A4* gene, IVS7-2A>G, c.1229C>T, and c.2168A>G are the top 3 most common deafness-related mutations in the Chinese population, including Taiwanese [11, 12, 13]. As another important deafness-related gene, mitochondrial gene *MT-RNR1*, a meta-analysis demonstrated pooled prevalence in the general population for *MT-RNR1* gene mutations (m.1494C>T, m.1555A>G, and m.7445A>G) was 2% (1%–4%) at 99%, which makes routine screening in high-risk Chinese populations justified [14]. More recently, m.12202T>C mutation is confirmed to be associated with non-syndromic deafness. The prevalence of m.12202T>C in Chinese hearing-loss patients is reported about 0.11% [15]. In addition, mutations in *GJB3* were originally identified to underlie an autosomal dominant form of NSHL in China. And c.538C>T in *GJB3* was previously considered to be responsible for high-frequency hearing loss [16]. However, recent studies showed that the incidence of the *GJB3* c.538C>T variant has a very low incidence (0.40%) in Chinese hearing-loss patients [17]. In our study, we still include *GJB3* c.538C>T variant in the detection system because screening for *GJB3* in the Chinese population is still necessary and cannot be removed hastily.

In China, the traditional audiological examination is applied in most medical institutions for screening deafness at present. However, this traditional examination is not suitable for detecting hereditary deafness [1, 18]. In recent years, more different methods are developed to detect mutations in NAHL-related genes, including classic polymerase chain reaction-restriction enzyme analysis (PCR-RFLP) [19], matrix-assisted laser desorption-ionization time-of-flight mass spectrometry (MALDI-TOF-MS) [20], gene sequencing, etc. However, due to the limitation of funds and equipment, it is impossible to carry out expensive genetic tests for hearing loss in primary hospitals in China. Therefore, it is necessary to develop an economical and convenient genetic testing method for early detection of hearing loss in primary hospitals.

In this study, we established a reliable gene-detection method based on a reverse dot blot assay combined with a flow-through hybridization technology platform that allows the simultaneous detection and genotyping of 13 hotspot mutations of 4 prominent hearing loss–related genes (*GJB2, GJB3, SLC26A4,* and *MT-RNR1*). The established system was applied in different PCR platforms and has the advantages of time- and money-saving.

## Methods

A total of 213 volunteers were collected from Changning Maternity and Infant Health Hospital. This study was approved by the ethics committee of Changning Maternity and Infant Health Hospital (CNFBLLJD-2021-01). Each participant has signed the informed consent. Human whole blood samples were obtained, and genomic DNA was extracted using Hybribio Genomic DNA Extraction Kit (Hybribio) according to the manufacturer's instructions. DNA concentration was measured by NanoDrop 2000 (Thermo Fisher Scientific). In addition, Chaozhou Hybribio Limited Corporation also provided 26 samples of different hearing loss gene mutations verified by gene sequencing. Among them, 13 samples were used for method reproducibility, and the other 13 were used for validation tests.

### Design of primers and probes

**Figure 1.**
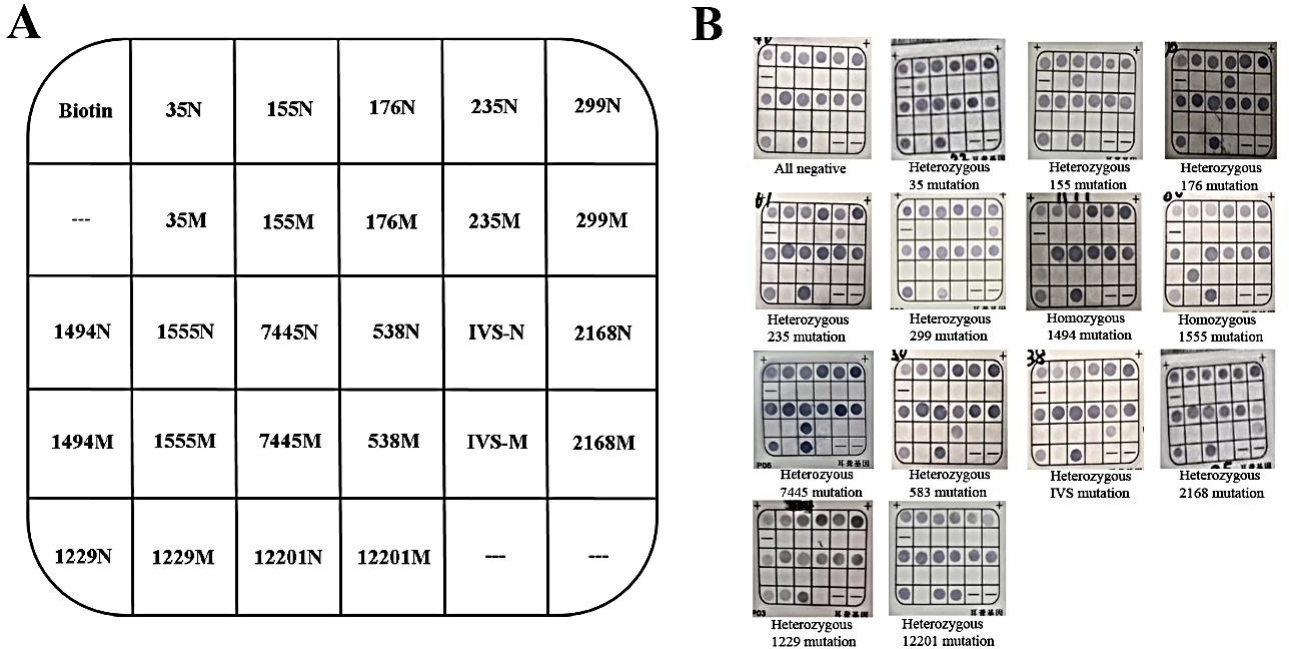
Schematic representation and results of hearing loss gene chip. **(A)** The schematic representation of the location of the probes dotted in the hearing loss gene chip used for the reverse dot blot assay. N represented the location of wild-type probes. M represented the location of mutant probes. **(B)** Part of the hybridization results of hearing loss gene chip, which included heterozygous, and homozygous gene mutations. (-) presented no probe at the corresponding position in the hybridization membrane.

The hearing loss–detection kit which was designed and made by Chaozhou Hybribio Limited Corporation included a PCR system as follows: one set of primers of *GJB2* was designed to amplify the *GJB2* gene, generating a 433 bp amplified fragment for detecting 5 common mutations including c.35delG, c.155_158del4, c.176_191del16, c.235delC, and c.299_300delAT. *GJB3* primers were designed to amplify the *GJB3* gene, generating 343 bp amplified fragments for detecting c.538C>T. And 3 sets of primers of *SLC26A4* were designed to amplify the *SLC26A4* gene, generating 549bp, 606bp, and 106bp amplified fragments for detecting 3 common mutations including IVS7-2A>G, c.1229C>T and c.2168A>G. Also, the 3 sets of primers of *MT-RNR1* were designed to amplify *MT-RNR1*gene, generating 381bp, 489bp, and 624bp amplified fragments for detecting 4 common mutations including m.1494C>T, m.1555A>G, m.7445A>G, and m.12201T>C. All probes were immobilized on a nylon membrane. **Figure 1A** shows their localization in the membrane. The detailed nucleotide sequence of the primers and probes are listed in **Tables 1 and 2**. Some of the primers were biotinylated (**Table 1**).

**Table 1.** The information on primers in the reaction system

**Name**	**Sequence (5′→3′)**
GJB2-F	(Biotin)-TAGTGATTCCTGTGTTGTGTG
GJB2-R	TTCTGGGTTTTTGATCTCCTC
GJB3-F	(Biotin)-CTCTTCCTCTACCTGCTGC
GJB3-R	TATTGCCTGGGTCTGGAT
SLC26A4-F1	(Biotin)-TTCACTGCTGGATTGCTCA
SLC26A4-R1	GTGTTAACCGTACATGTTCTGC
SLC26A4-F2	(Biotin)-CTCTCAGATGGTATGGCGTC
SLC26A4-R2	TCCTTCATTACTGATTCCTTGTC
SLC26A4-F3	(Biotin)-GTTCTTTGACGACAACATTAG
SLC26A3-R3	AATGGAACCTTGACCCTCTT
mtDNA-F1	(Biotin)-ATGAGGTGGCAAGAAATG
mtDNA-R1	TTGGCTAAGGTTGTCTGGTA
mtDNA-F2	(Biotin)-GATGCATACACCACATGAAAC
mtDNA-R2	TGAGTGTTAGGAAAAGGGCA
mtDNA-F3	(Biotin)-CACAATGGGGCTCACTCA
mtDNA-R3	ACGAACAATGCTACAGGGAT

**Table 2.** The information of probes in the hearing loss gene chip

**Name**	**Sequence (5′→3′)**
1494-PN	CGTCACCCTCCTCAAG
1494-PM	CCGTCACTCTCCTCAAG
1555 PN	AGAGGAGACAAGTCGTAAC
1555 PM	GAGGAGGCAAGTCGTAA
7445 PN	ACATAAAATCTAGA-CAAAAAAGGA
7445 PM	CATAAAATCTAGGCAAAAAAGG
12201 PN	GACCCCTTATTTACCGAGAA
12201 PM	CCCCTTACTTACCGAGA
G2-35 PN	GTTCACACCCCCCAGGA
G2-35 PM	AATGGAACCTTGACCCTCTT
G2-155 PN	GGTGTTGCAGACAAAGTCG
G2-155 PM	CGACTTTGCAACACCC
G2-176 PN	GCACACGTTCTTGCAGCC
G2-176 PM	CAGCCAGCTACGATCAC
G2-235 PN	AGCTGCAGGGCCCATA
G2-235 PM	TATGGGCCTGCAGCT
G2-299 PN	TTCTTCTCATGTCTCCGGT
G2-299 PM	ACCGGAGACGAGAAGAA
G3-538 PN	ACATTGCCCGACCTAC
G3-538 PM	ACATTGCCTGACCTACC
IVS7-2 PN	GTTTTATTTCAGACGATAATTGC
IVS7-2 PM	TGTTTTATTTCGGACGATAAT
1229 PN	CTGCTCTTTCCCGCACGG
1229 PM	CTCTTTCCCGCATGGCC
2168 PN	CGGTCCATGATGCTATA
2168 PM	GTCCGTGATGCTATACT

### PCR amplification

The PCR amplification was carried out in strict accordance with the manufacturer's procedure [21]. Briefly, the PCR amplification system contained a total reaction volume of 30 μl containing 50 ng of DNA template, 28 μl 2 × GC buffer, 4 mmol/L MgCl_2_, 0.2 mmol/L of each primer (**Table 1**), 0.2 mmol/L of each dNTP, and 2.5 units of HotStart DNA polymerase (Hybribio) in a MJ Mini Personal Thermal Cycler (Bio-RAD Company) and the PCR reaction was performed with an initial 9 min denaturation at 95 °C, 40 cycles of 95 °C for 30 s, 55 °C for 30 s, 72 °C for 60 s, and a final extension at 72 °C for 5 min. The PCR amplification products were subsequently denatured and subjected to hybridization.

#### Flow-Through hybridization

Hybridization reactions were carried out using HMM2I (Chaozhou Hybribio Limited Corporation) as in our previous study [22]. The details of the flow-through hybridization method are as follows: first, directly incubate the PCR products (target molecules) with the membrane fibers that contain the immobilized probes. Then, the complementary molecules are retained on the membrane by the formation of duplexes with a probe. After a careful wash, the streptavidin–horseradish peroxidase conjugate binds to biotinylated PCR products, and a certain substrate (nitro-blue tetrazolium-5-bromo-4-chloro-3-indolylphosphate, BCIP) is added to detect the hybrids. The results were interpreted by direct visualization [22].

In each assay, three kinds of quality controls were used, including blank control, negative control, and positive control. The blank control was ddH2O, the negative control was provided by Chaozhou Hybribio Limited Corporation, and the positive control was purchased from Guangzhou BDS Biological Technology Co., Ltd.

### Reproducibility of the method

Two different batches of reagents and instruments from different manufacturers (GeneAmp7500 of PE Applied Biosystems and MJ Mini Personal Thermal Cycler of Bio-RAD Company) were used to analyze 13 samples with different genotypes of hearing loss to validate the reproducibility and precision of this assay. Each sample detection was performed five times. Statistical analysis was carried out for each sample.

### DNA sequencing

The PCR amplification products of 13 mutations in 4 hearing loss–related genes, including *GJB2*, *GJB3*, *SLC26A4*, and *MT-RNR1*, were sequenced by ABI 3700 automated sequencer as in our previous research [23].

### Data analysis and statistics

All data were analyzed by SPSS version 17.0 statistical software (SPSS Inc.). The crude percent agreement between the reverse dot blot assay and the DNA sequencing was the percentage of samples with identical results by both methods. Absolute agreement and Cohen's kappa statistics were applied to assess the consistency of the results of the two detection methods. *P* values of <0.05 were considered statistically significant.

## Results

### Hybridization results of the hearing loss gene chip

The visual results of the hybridization reactions of the hearing-loss genes are shown in **Figure 1**. The colored dot at a specific location in the membrane represents the corresponding gene mutation status. Thirteen mutations of hearing-loss genes are given in **Figure 1B**. After the hybridization reactions, all normal control dots on the chip membrane would be colored.

### Validation and application of the assay

Thirteen samples with different mutation sites of hearing-loss genes were detected at least five times in order to evaluate the reproducibility of the reverse dot blot assay. All the wild-type and mutant dot blots exhibited the same depth of color each time. There were no differences among them.

In a verification test, we chose another 13 samples with different genotypes of mutation sites of hearing-loss genes. All these genomic DNA samples were detected by both DNA sequencing and our reverse dot blot assay, and the results were consistent with each other. Moreover, the reverse dot blot assays were performed by two operators with different batches of reagents and two different PCR instruments (GeneAmp7500 and MJ Mini Personal Thermal Cycler), and the results were then also consistent.

**Figure 2.**
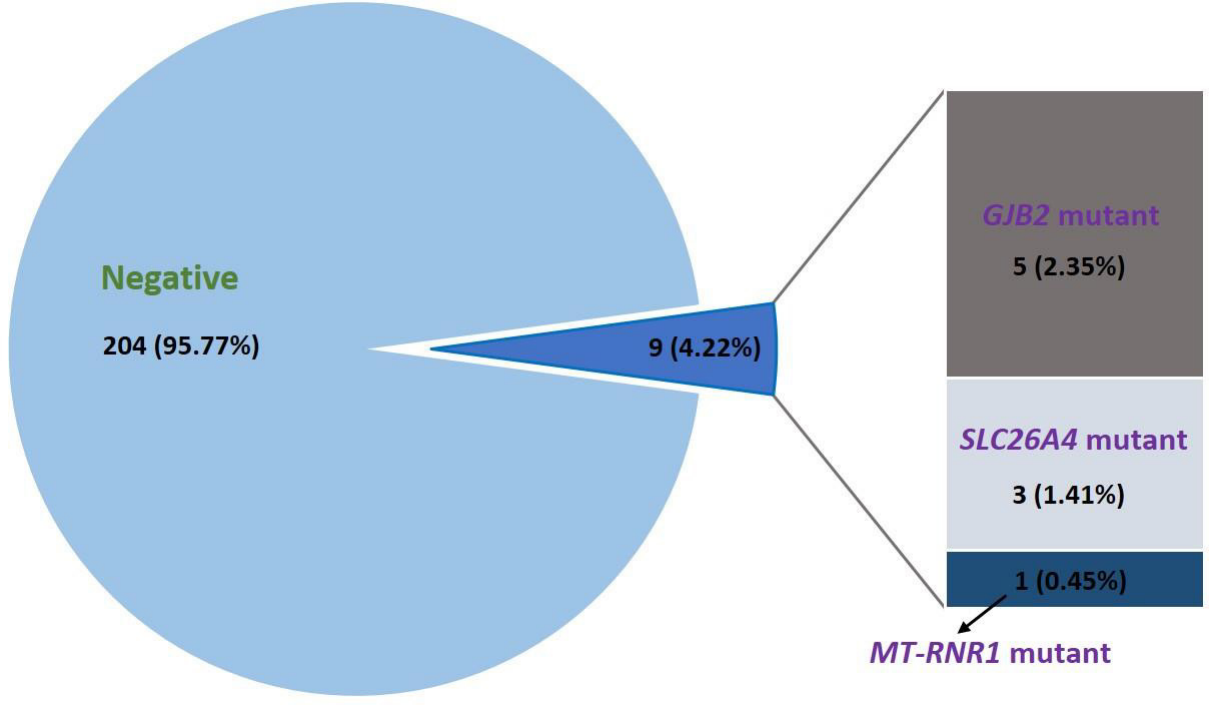
Detection of deafness gene mutations in 213 volunteers.

After establishing the hearing loss gene mutation detection system, we applied the detection system in a primary hospital. A total of 213 genomic DNA samples were collected and performed the hearing loss gene mutation analysis. Of the 213 volunteers, a total of 9 participants carried hearing loss gene mutate alleles, which indicates a positive rate of 4.23% (**Figure 2**). All of the 9 individuals are carriers of single-gene heterozygous mutations in our research. As shown in **Table 3**, 5 participants carried mutations in *GJB2* (5/213, 2.35%), 3 in *SLC26A4* (3/213, 1.41%), and 1 in m.1555A>G *MT-RNR1* (1/213, 0.47%). Of those, 4 subjects carried c.235delC *GJB2* variation, 1 participant carried c.299_300delAT *GJB2* mutation, 2 participants revealed IVS7-2A>G *SLC26A4* mutation, and 1 carried c.2168A>G *SLC26A4* mutation. However, the *GJB3* mutation c.538C>T was not detected in any genomic DNA sample.

**Table 3.** The genotypes of nine individuals with hearing loss gene mutation

***GJB2***	**n**	***GJB3***	**n**	***SLC26A4***	**n**	***mtDNA***	**n**
c.35delG	0	c.538C>T	0	IVS7-2A>G	2	m.1494C>T	0
c.155_158del4	0			c.1229C>T	1	m.1555A>G	1
c.176_191del16	0			c.2168A>G	0	m.7445A>G	0
c.235delC	4					m.12201T>C	0
c.299_300delAT	1						

## Discussion

More than 50% of hearing-loss cases have genetic causes. The 2014 American College of Medical Genetics and Genomics (ACMG) guidelines have underlined the importance of an etiological diagnosis for hearing loss, in which genetic testing is recommended to be included in the workup of patients with NSHL [24]. In China, it is very important to establish a convenient, economical, rapid, and effective genetic-detection method for NSHL diagnosis in primary hospitals.

Here, we established a new gene-detection system by using a reverse dot blot assay technique. It could simultaneously detect 13 hot mutations of hearing loss–related genes, enabling effective analysis of the genetic information in a sample. The key feature of this system is the use of the common PCR instrument to amplify hearing loss–related genes followed by a specific reverse dot blot assay with site-specific probes to identify the 13 mutation sites. Each mutation site was detected by a specific probe. The reverse dot blot assay system ensures that all the 13 mutation sites can be identified in a single reverse dot blot membrane.

According to the reported studies, various methods were used to detect hearing-loss genes, such as probe melting curve [25], microarray [26], amplification refractory mutation system PCR [27], denaturing gradient gel electrophoresis (DGGE) [28, 29], and denaturing high-performance liquid chromatography (DHPLC) [30]. However, these methods have the disadvantages of being expensive or not being accurate enough. At present, genetic screening for hearing loss is not widely available in primary hospitals in China. Although quantitative PCR is used in some institutions or hospitals to detect mutations of hearing loss–related genes, the equipment is expensive and cannot be widely used in primary hospitals. Our detection method does not require special equipment. In general, our reverse dot blot assay has the following advantages: first, compared with other methods that can detect only 9 mutation sites, this method can detect 13 common mutation sites of 4 hearing loss–related genes simultaneously. It is necessary to increase the screening of deafness gene mutation sites in primary hospitals. Second, a gene-detection system can be easily performed, as the PCR amplification can be set up in all instruments. It will also possible to establish automatic detection in the future. Third, the flow through the hybridization platform can effectively shorten the hybridization time by about 4.5 h, from 6 h to 1.5 h [22]. The reverse dot blot assay could process 16 samples and get the detection result within 1.5 h in a single run. The sample size of each run of our detection system is larger than that of other methods, leading to time-saving. However, the reverse dot blot assay also has some disadvantages: while 13 mutations can be detected by this method, other known–unknown mutations have not yet been detected. In the future, we can easily add more detection sites to our detection system.

In fact, we applied the gene-detection system in a primary hospital in Shanghai, China. The results of hearing loss gene mutation analysis with 213 genomic DNA samples indicated a positive rate of 4.23%. The mutation distribution of deafness genes in these samples was consistent with those in the Chinese population. The results indicate that this method is feasible to be applied in Chinese primary hospitals. Nevertheless, this study has some limitations. First, this detection method is only used to detect 13 mutation sites in 4 common hearing-loss genes in the Chinese population, and it does not cover all mutation sites related to hearing-loss genes. Therefore, even if the test result is negative, it cannot be ruled out that the subject has other mutation sites related to hearing-loss genes. Second, test results can only assist in making a clinical diagnosis. Clinicians must make a diagnosis of patients in combination with other clinical examinations, medical history, etc. Moreover, in the present study, the sample size is relatively small, with only more than 200 cases. Therefore, in follow-up studies, we will expand the sample size to further explore the distribution of deafness gene mutation sites in the Chinese population. Despite this, this method still has great clinical application prospects. It can not only be used as a screening method for high-risk populations, but can also be widely used in primary hospitals.

## Conclusion

In conclusion, we have established a novel gene-detection system based on the reverse dot blotting assay technique to simultaneously detect 13 mutation sites in 4 hearing loss–related genes. The reverse dot blot assay ensures their liability and visualization of this gene-detection system. The detection system is widely applicable to common PCR instruments, and it is easy to manipulate and is of low cost, high performance, and high accuracy. It has a wide clinical application prospect.
